# Cannabidiol impairs neural tube closure in mouse whole embryo culture

**DOI:** 10.1002/bdr2.2013

**Published:** 2022-04-13

**Authors:** Yosuf Gheasuddin, Gabriel L. Galea

**Affiliations:** ^1^ Developmental Biology and Cancer UCL GOS Institute of Child Health London UK

**Keywords:** Cannabidiol, exencephaly, mouse, neural tube, spina bifida, whole embryo culture

## Abstract

**Background:**

Cannabidiol (CBD) is a nonpsychoactive constituent of cannabis widely available as a dietary supplement. Previous reports that it impairs the retinoid, sonic hedgehog, and folate metabolism pathways raise concern that it may impair closure of the embryonic neural tube (NT), producing NT defects including spina bifida and exencephaly.

**Methods:**

We undertook teratogenicity testing of CBD in mouse whole embryo culture.

**Results:**

At concentrations that do not diminish embryo viability, growth, or axial rotation, CBD dose‐dependently impairs cranial NT closure, increasing the proportion of embryos that develop exencephaly. It concomitantly diminishes closure of the spinal NT, the posterior neuropore (PNP), producing longer neuropores at the end of culture which is a hallmark of spina bifida risk. Exposure to CBD does not disrupt the formation of long F‐actin cables in surface ectoderm cells flanking the PNP or folding of the neuroepithelium at predictable hinge points. At the cellular level, CBD exposure does not alter proliferation or apoptosis of the spinal neuroepithelium.

**Discussion:**

Thus, CBD acts selectively as a neuroteratogen predisposing to spina bifida and exencephaly in mouse whole embryo culture at exposure levels not associated with overt toxicity. Large‐scale testing of CBD's effects on NT closure, particularly in at‐risk groups, is warranted to inform its marketing to women of childbearing age.

## 1 INTRODUCTION

Cannabidiol (CBD) is a nonpsychoactive constituent of cannabis available in licensed medications, dietary supplements, and topical formulations. Its biological effects are at least partly mediated by its partial agonist action on cannabinoid receptors (Zagzoog et al., 2020). However, it also has less‐understood effects on other signaling pathways. In *Dictyostelium*, it reduces levels of tetrahydrofolate, a component of folate one‐carbon metabolism (Perry et al., 2020). In zebrafish larvae, CBD treatment alters the transcription of genes involved in retinoic acid signaling (Pandelides, Aluru, Thornton, Watts, & Willett, 2021). In mice, both folate metabolism (Burren et al., 2008) and retinoic acid signaling (Li et al., 2018) promote closure of the neural tube (NT), the embryonic precursor of the central nervous system. NT closure initiates at the hindbrain‐cervical boundary before zippering posteriorly to gradually close the posterior neuropore (PNP) and anteriorly toward the brain, where two additional closure‐initiating points form in mice (Nikolopoulou, Galea, Rolo, Greene, & Copp, 2017). Failure of NT closure causes NT defects (NTDs) including spina bifida and exencephaly/anencephaly in 1:1,000 births globally (Zaganjor et al., 2016). Given the popularity of CBD products in several countries (Bhamra, Desai, Imani‐Berendjestanki, & Horgan, 2021; de Albuquerque Britto, Angelo Mendes, Tenorio, Rolim, & Junior, 2021; Lachenmeier et al., 2019) and its potential to disrupt pathways involved in NT closure, we subjected CBD to teratogenicity testing in mouse whole embryo culture.

CBD teratogenicity testing in chick embryos in ovo has previously been reported, diminishing embryo growth and viability at exposure concentrations between 20 and 50 μM (Gustafsson & Jacobsson, 2019). No structural malformations were observed in treated embryos, but CBD was not administered in the 35–50 hr incubation window during which the chick NT closes (Van Straaten, Janssen, Peeters, Copp, & Hekking, 1996). In vivo CBD teratogenicity testing of orally administered purified CBD during preclinical licensing trials has previously been shown to cause embryo/fetal developmental delay in rats and rabbits at high doses, but direct analysis of NT closure was not included (FDA, 2018; Huestis et al., 2019). Mouse embryos initiate NT closure on embryonic day (E)8.5 and complete it by E10.5. This period of development is amenable to experimentation in whole embryo culture, avoiding potentially species‐specific maternal metabolism. Many agents known to cause NTDs in humans diminish NT closure in mouse whole embryo culture including valproic acid (Hughes, Greene, Copp, & Galea, 2018), fumonisin (Sadler et al., 2002), and retinoic acid (Santos‐Guzman et al., 2003).

Here, we treated mouse embryos with CBD in whole embryo culture to assess its effects on embryo development and progression of NT closure. General cytotoxic effects were assessed by counting mitotic and apoptotic neuroepithelial cells in the open PNP. We find that CBD selectively diminishes both cranial and spinal NT closure at concentrations that do not impair embryo development.

## 2 METHODS

### 2.1 Embryo culture and treatments

C57Bl/6J mice were bred in‐house and mated when at least 6 weeks old. Studies were performed under project and personal licenses regulated by the UK Animals (Scientific Procedures) Act 1986 and the Medical Research Council's Responsibility in the Use of Animals for Medical Research (1993). Pregnant female mice were sacrificed by cervical dislocation.

CBD was purchased from Sigma‐Aldrich (Cat. no. 90899‐1ML, 10 mg/ml solution in ethanol, >98.5% purity, CAS number 13956‐29‐1). CBD solution was diluted in ethanol vehicle and added at the start of embryo culture at a maximum concentration of 0.1% ethanol in rat serum. Embryo culture was performed as previously described (Hughes et al., 2018). Mice were mated overnight and the morning a plug was found being considered E0.5. Pregnant females were sacrificed in the morning of E8.5 (~6 somites at the start of culture) and their embryos were cultured for 24–30 hr. Each litter was divided into two size‐matched groups of 3–4 embryos per group and a coin toss was used to determine which would receive CBD or vehicle treatment.

At the end of culture, embryos were assessed for yolk sac circulation and an active heartbeat (present in all embryos). Embryos were dissected out of their extraembryonic membranes and fixed in 4% paraformaldehyde overnight.

### 2.2 Whole‐mount staining and microscopy

Embryo whole‐mount staining as previously described (Galea et al., 2017). Primary antibodies used were mouse anti‐phospho‐histone H3 (S10, Cell Signaling Technology antibody #9701) and rabbit anti‐cleaved caspase‐3 (Cell Signaling Technology antibody #9661) and detected with Alexa Fluor™ conjugated secondary antibodies. F‐actin was labeled with Alexa Fluor™‐647 conjugated phalloidin. Images were captured on a Zeiss Examiner LSM880 confocal using a 10×/NA0.5 Plan Apochromat water immersion objective. Images were processed and visualized as 3D or maximum projections in Fiji (Schindelin et al., 2012).

Stereoscope images were captured using a Leica DFC490 camera mounted on a Zeiss Stemi SV‐11 stereomicroscope. Dorsally oriented PNP images were captured to analyze PNP length and embryo lateral images were captured to measure dorsal length as a curved line from the otic vesicles to the caudal tip using Fiji.

### 2.3 Statistical analysis

The litter was considered the unit of measure for continuous data from brightfield images (yolk sac diameter, somites, dorsal length, head length, and PNP length). The mean was calculated for the 3–4 embryos cultured in each condition and compared using *t* tests paired by litter. For quantifications based on confocal images (F‐actin cable length, surface ectoderm, pHH3, and cleaved caspase‐3 staining), a vehicle and CBD‐treated embryo were randomly selected from each litter and compared with unpaired *t* tests.

For binary (cranial NT closure) and categorical (turning score) variables, the individual embryo was the requisite unit of measure and groups were compared using Fisher's exact test.

*p* < .05 was considered statistically significant.

## 3 RESULTS

### 3.1 CBD does not impair development mouse embryo development in culture

**FIGURE 1:**
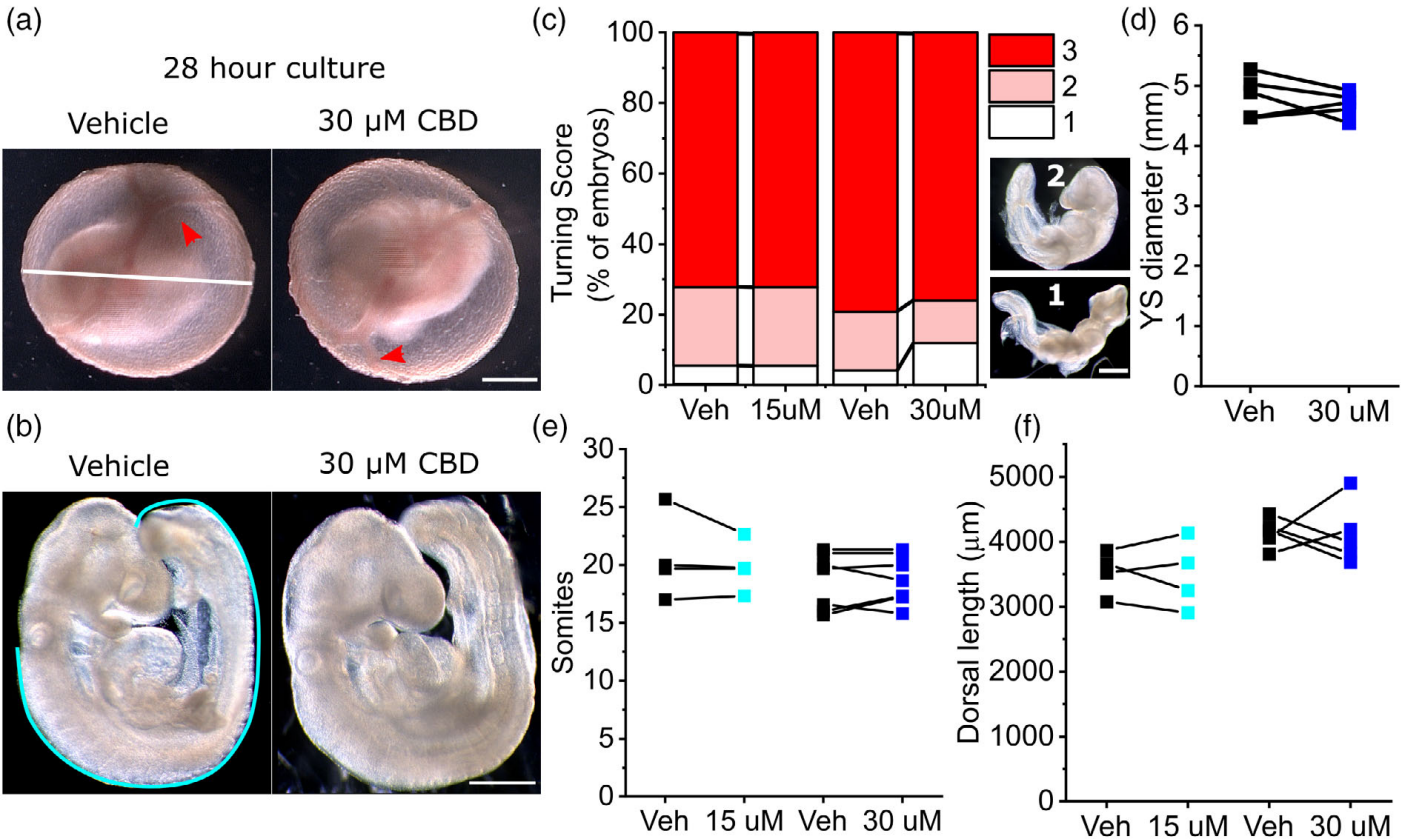
CBD does not significantly impair mouse embryo growth in culture. (a) Representative vehicle (ethanol) and 30 μM CBD treated litter embryos at the end of culture. Red arrows show yolk sac vasculature. The white line indicates yolk sac diameter, quantified in panel (d). (b) Vehicle and CBD‐treated embryos showing normal axial rotation. The Cyan line indicates dorsal length measured from the otic pit to the end of the tail. (c) Semi‐quantitative analysis of axial rotation (1–3) comparing vehicle versus littermate CBD‐treated embryos. Illustrative embryos with incomplete turning are shown (scores 1 and 2), whereas the embryos in (b) have completed turning (score 3). Data are the aggregate of four litters in the 15 μM group and five litters in the 30 μM group. (d–f) Quantification of (d) yolk sac diameter, (e) somite pairs, and (f) embryonic dorsal length in vehicle (Veh) and littermate CBD‐treated embryos. Points represent the mean of 3–4 embryos per condition and lines connect paired points from the same litter. Scale bars = 1 mm. No comparisons were significantly different between vehicle and CBD‐treated groupings. CBD, Cannabidiol

Measured concentrations of CBD in widely available formulations range from 0.1 (318 μM) to >650 mg/ml (2 M), with marketed concentrations differing from measured values by tenfold on average (Bonn‐Miller et al., 2017). Previous in vitro studies have tested the cellular effects of CBD at concentrations up to 50 μM (Pagano et al., 2020). We tested the effect of 15 or 30 μM CBD in mouse whole embryo cultures. Between E8.5 and E9.5, embryos develop prominent yolk sac circulation and undergo axial rotation (Figure 1a,b). At the concentrations tested, CBD treatment does not significantly impact embryonic axial rotation (Figure 1c), semi‐quantitively assessed using a previously described turning score (Culshaw, Savery, Greene, & Copp, 2019). Expansion of the yolk sac is also not impacted by CBD exposure (Figure 1d, only the 30 μM and associated vehicle groups were quantified). CBD treatment does not diminish the increase in either somite pairs or dorsal length, which represent direct measurements of embryo growth during culture (Figure 1e,f).

### 3.2 CBD impairs NT closure

**FIGURE 2:**
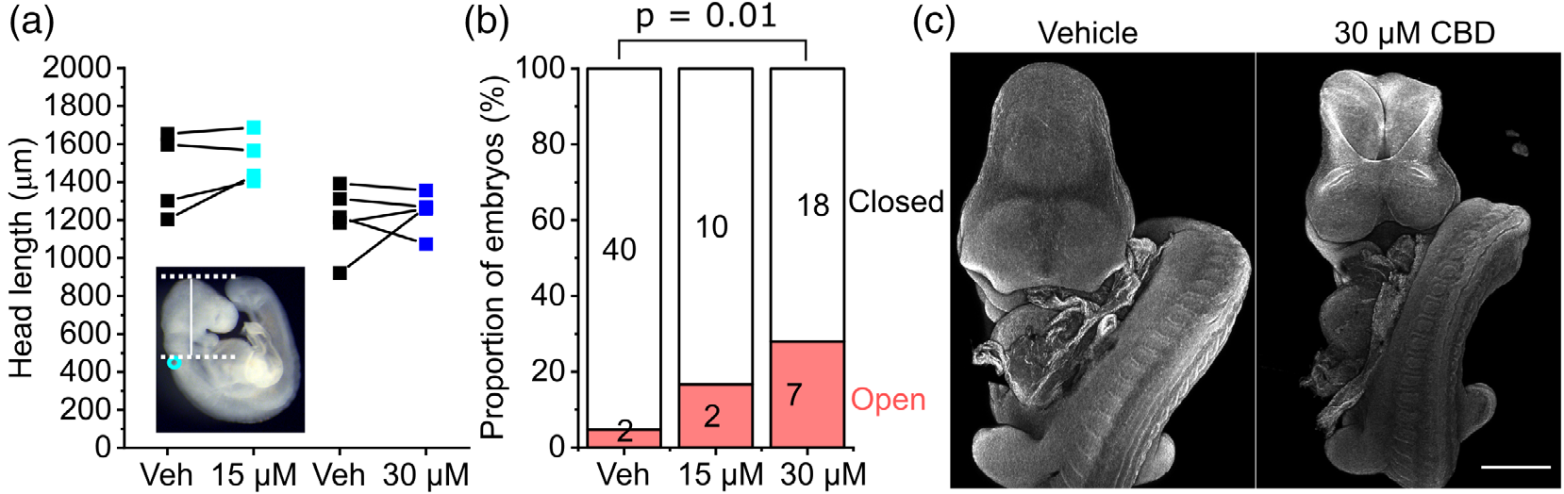
CBD impairs cranial neural tube closure. (a) Quantification of head length as indicated by the vertical white line in the inset (cyan circle shows the otic pit). Points represent the mean of 3–4 embryos per condition and lines connect paired points from the same litter. Neither comparison between vehicle and CBD treatments were significantly different. (b) Quantification of the proportion of embryos with >17 somites at the end of culture which had open or closed cranial neuropores. The number of embryos in each group is indicated. (c) Representative confocal images showing a vehicle‐treated embryo with closed cranial NT and a CBD‐treated littermate with exencephaly. Scale bar = 500 μm. CBD, Cannabidiol

Mouse embryos normally complete closure of the cranial NT by the 17‐somite stage (Maniou et al., 2021), after which the head rapidly expands. CBD treatment did not significantly impact the expansion of the embryonic head in embryos that successfully closed their NT (Figure 2a). However, CBD dose‐dependently impaired successful closure of the cranial NT, causing exencephaly (Figure 2b,c). The proportion of embryos that achieved 17 somites by the end of culture and still had open cranial NT was significantly greater when exposed to 30 μM CBD compared with littermate vehicle controls (30 μM group: vehicle = 0/25, CBD = 7/25 embryos >17 somites with open cranial NT, *p* = .01 by Fisher's exact test).

**FIGURE 3:**
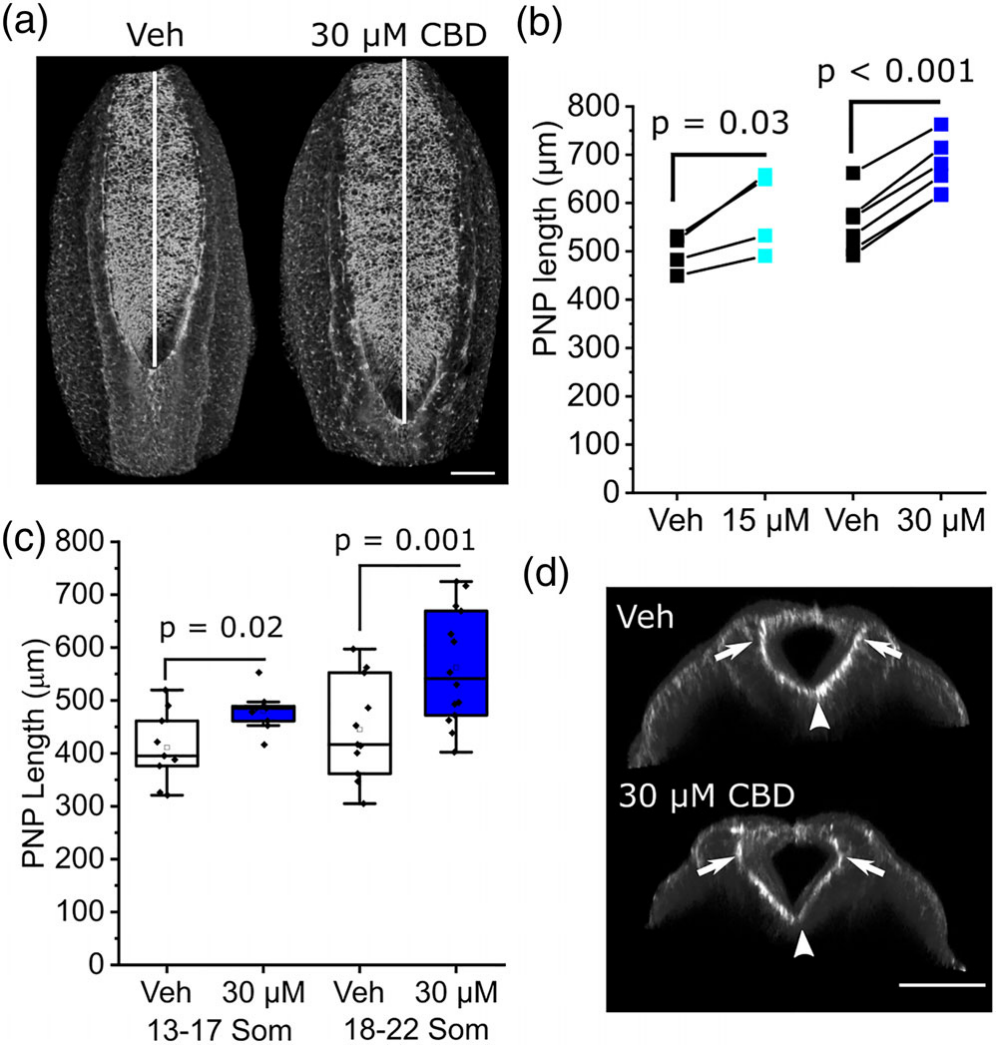
CBD diminishes closure of the spinal NT. (a) Representative 3D projections showing F‐actin labeled PNPs of a vehicle and littermate CBD‐treated embryos. Vertical lines indicate PNP length. (b) Quantification of PNP length. Points represent the mean of 3–4 embryos per condition and lines connect paired points from the same litter. (c) Quantification of PNP length in vehicle and 30 μM CBD treated embryos which reached the indicated somite stages at the end of culture in. Points represent individual embryos. (d) Optically resliced confocal images showing the median hinge point (arrow heads) and dorsolateral hinge points (arrows) in the PNP of a vehicle and littermate CBD‐treated embryo. Scale bars = 100 μm. CBD, Cannabidiol; NT, neural tube; PNP, posterior neuropore

Closure of the spinal PNP continues until the ~30 somite stage (~E10.5), beyond the time frame of cultures described here. PNP length is commonly used as a surrogate measure of susceptibility to failure of closure, causing spina bifida. CBD treatment diminishes PNP shortening, producing significantly longer PNPs than vehicle‐treated littermates by the end of the culture period at both concentrations tested (Figure 3a,b). Longer PNPs are evident in embryos that reached 13–17 somites by the end of culture and were more marked in embryos that developed 18–22 somites (Figure 3c). Characteristic bending of the PNP neuroepithelium at the median and dorsolateral hinge points can still be observed in CBD‐treated embryos (Figure 3d).

**FIGURE 4:**
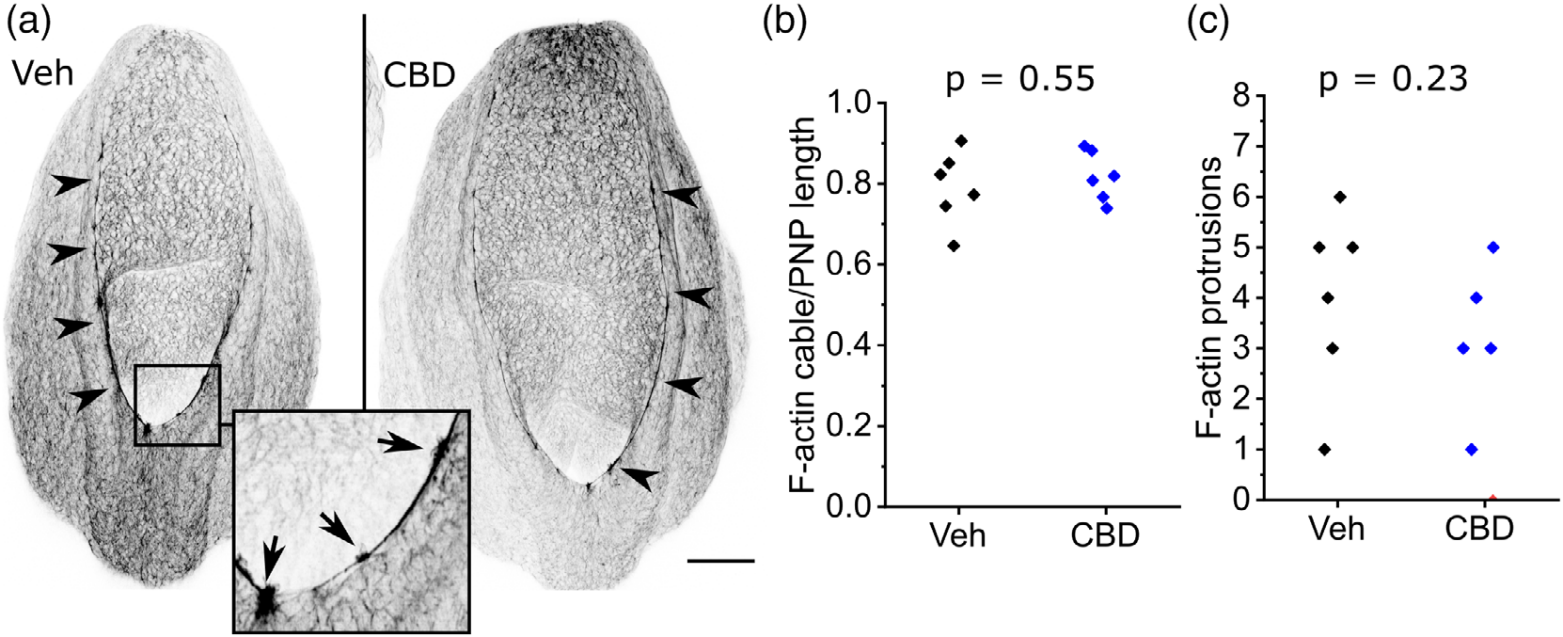
CBD treatment does not diminish surface ectoderm F‐actin cables or zippering protrusions. (a) Representative maximum projections of F‐actin labeled (inverted gray LUT) PNPs from a vehicle and littermate CBD‐treated embryo. Arrowheads denote supracellular F‐actin cables lining the neural folds. Arrows in the inset indicate F‐actin‐rich cellular protrusions. Scale bar = 100 μm. (b, c) Quantification of (b) F‐actin cable length as a proportion of total PNP length and (c) the number of cellular protrusions along the cables in vehicle and 30 μM CBD‐treated embryos. Points represent individual embryos. CBD, Cannabidiol; PNP, posterior neuropore

Shortening of the PNP is achieved by zippering, whereby surface ectoderm cells extend F‐actin‐rich protrusions that meet their contralateral partner across the midline (Rolo et al., 2016). Surface ectoderm cells also form long actomyosin cables which run along the neural folds: these cables are shorter in embryos with diminished PNP closure due to valproate treatment (Hughes et al., 2018). Neither the actomyosin cables nor the presence of their associated F‐actin‐rich protrusions is significantly impacted by CBD treatment (Figure 4a–c).

### 3.3 CBD does not alter neuroepithelial proliferation or apoptosis

**FIGURE 5:**
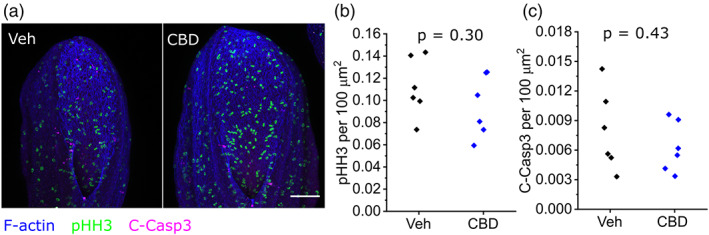
CBD treatment does not alter neuroepithelial proliferation or apoptosis. (a) Representative maximum projections of whole‐mount labeled PNPs from a vehicle and littermate CBD‐treated embryo. Scale bar = 100 μm. (b, c) Quantification of (b) phospho‐histone H3 and (c) cleaved caspase‐3 labeled cells normalized to PNP area in vehicle and 30 μM CBD‐treated embryos. Points represent individual embryos. CBD, Cannabidiol; PNP, posterior neuropore

CBD treatment has been reported to diminish proliferation and induce apoptosis of cultured cells (Alves, Amaral, Teixeira, & Correia‐da‐Silva, 2021; Hamad & Olsen, 2021; Sainz‐Cort, Muller‐Sanchez, & Espel, 2020). We assessed proliferation by counting phospho‐histone H3 positive cells, and apoptosis by counting cleaved caspase‐3 positive cells, in the exposed neuroepithelium of the open PNP. Treatment with 30 μM CBD, sufficient to impair NT closure, does not significantly alter proliferation or apoptosis 24 hr after initiation of whole embryo culture (Figure 5a–c).

## 4 DISCUSSION

Impairment of NT closure in cultured mouse embryos exposed to CBD adds to concerns regarding this compound's teratogenic potential. Previous teratology studies observed embryo/fetal mortality or developmental delay (Huestis et al., 2019) and in vivo administration of cannabinoids including CBD causes eye and palate malformations in mice (Fish et al., 2019). Cannabis use has been epidemiologically linked to major congenital malformations including NTDs in humans (Reece & Hulse, 2019a, 2019b) and its use is associated with higher exposure to CBD in other forms (Dunbar et al., 2022). Not all cannabinoids exert equivalent effects on embryo development. For example, various cannabinoids, but not CBD, arrest the development of peri‐implantation mouse embryos (Paria, Das, & Dey, 1995). Controlled teratogenicity testing is therefore necessary to inform the potential contribution of individual cannabinoids to congenital malformations such as NTDs.

In the present study, CBD exposure minimally impacted mouse embryo development in whole embryo culture. This contrasts with previous studies which identified developmental delays in embryos of rats and rabbits treated with CBD in vivo (Huestis et al., 2019). Possible explanations for this discrepancy include the effects of CBD on maternal cells, such as its reduction of endometrial differentiation involved in decidualization (Almada et al., 2020). Species differences may also be important. Toxicity has previously been reported in zebrafish exposed to low micromolar concentrations of CBD (Ahmed, Amin, Shah, & Ali, 2018; Carty, Thornton, Gledhill, & Willett, 2018) as well as in chick embryos chronically exposed following in ovo injection (Gustafsson & Jacobsson, 2019). As well as whole‐organism assessment of development progression, we undertook cell‐level analysis of proliferation and apoptosis in the exposed spinal neuroepithelium, corroborating the lack of toxic effects of CBD in mouse embryos. This is consistent with a previous report that in vivo administration of a synthetic cannabinoid does not markedly alter cell death in the cranial neural folds of early mouse embryos (Fish et al., 2019).

We document that CBD has direct neuroteratogenic effects on progression of both cranial and spinal NT closure in mice. Effects of CBD on neuron function and behavior have previously been studied in zebrafish (Carty et al., 2018; Carty et al., 2019; Kanyo et al., 2021), but this species does not close its NT in the same way as mammals do. The mechanisms by which CBD impairs the progression of NT closure have not been identified here and their delineation is likely to require substantial future work. The mechanisms by which it impairs cranial and spinal NT closure may be shared, or region‐specific. In the PNP, we exclude potential mechanisms including substantial effects of CBD on bending of the neuroepithelium or assembly of cytoskeletal structures including surface ectoderm F‐actin cables. Effects of CBD on other cellular behaviors potentially relevant to NT closure include its suppression of migration (Ramer et al., 2012), autophagy (Alves et al., 2021), and DNA methylation (Wanner, Colwell, Drown, & Faulk, 2020). Exencephaly has previously been observed in fetuses of mice treated in vivo with a synthetic cannabinoid, HU‐210 (Fish et al., 2019), suggesting the effects observed are relevant to other molecules in this class. Additionally, it is likely that CBD exposure may interact with both environmental factors, such as folate deficiency, and genetic predispositions to NTDs. A “conditional teratogen” effect has recently been reported for another cannabinoid, Δ9‐tetrahydrocannabinol, which causes CNS malformations including holoprosencephaly in a mutant mouse model of diminished sensitivity to Sonic hedgehog signaling (Lo, Hong, Szutorisz, Hurd, & Krauss, 2021).

Gene–gene and gene–environment interactions substantially contribute to NTDs in humans. Environmental or nutritional risk factors for NTDs continue to be identified, in part thanks to systematic testing in mouse models (Kakebeen & Niswander, 2021). Our observation of NTDs in cultured mouse embryos exposed to CBD should prompt systematic neuroteratogenicity testing including in models of increased NTD risk, targeted analysis of underlying mechanisms, and cautionary advice on its consumption to women intending to become pregnant.

## CONFLICT OF INTEREST

The authors declare no conflicts of interest.

## AUTHOR CONTRIBUTIONS

Both authors performed experiments and analyzed data. Gabriel L. Galea drafted the manuscript. Both authors approved the final manuscript.

## Data Availability

The data that support the findings of this study are available from the corresponding author upon reasonable request.
